# Sterile activation of RNA-sensing pathways in autoimmunity

**DOI:** 10.1093/jmcb/mjae029

**Published:** 2024-08-14

**Authors:** Jiaxin Li, Junyan Zhu, Hui Yang, Fajian Hou

**Affiliations:** Key Laboratory of RNA Science and Engineering, Shanghai Institute of Biochemistry and Cell Biology, Center for Excellence in Molecular Cell Science, Chinese Academy of Sciences, University of Chinese Academy of Sciences, Shanghai 200031, China; Key Laboratory of RNA Science and Engineering, Shanghai Institute of Biochemistry and Cell Biology, Center for Excellence in Molecular Cell Science, Chinese Academy of Sciences, University of Chinese Academy of Sciences, Shanghai 200031, China; Department of Neurosurgery, Huashan Hospital, Institute for Translational Brain Research, MOE Frontiers Center for Brain Science, Shanghai Medical College, Fudan University, Shanghai 200032, China; Shanghai Key Laboratory of Brain Function Restoration and Neural Regeneration, Huashan Hospital, Shanghai Medical College, Fudan University, Shanghai 200032, China; Key Laboratory of RNA Science and Engineering, Shanghai Institute of Biochemistry and Cell Biology, Center for Excellence in Molecular Cell Science, Chinese Academy of Sciences, University of Chinese Academy of Sciences, Shanghai 200031, China; Key Laboratory of Systems Health Science of Zhejiang Province, School of Life Science, Hangzhou Institute for Advanced Study, University of Chinese Academy of Sciences, Hangzhou 310024, China

**Keywords:** innate immune responses, RNA sensors, endogenous RNAs, autoimmunity

## Abstract

RNA-sensing pathways play a pivotal role in host defense against pathogenic infections to maintain cellular homeostasis. However, in the absence of infection, certain endogenous RNAs can serve as the activators of RNA-sensing pathways as well. The inappropriate activation of RNA-sensing pathways by self-ligands leads to systemic inflammation and autoimmune diseases. In this review, we summarize current findings on the sterile activation of RNA sensors, as well as its implications in autoimmunity, inflammatory diseases, and therapeutics.

## Introduction

The mammalian innate immune system relies on a diverse range of pattern recognition receptors (PRRs) capable of detecting pathogen-associated molecular pattern (PAMP) and danger damage-associated molecular pattern (DAMP) signals. These receptors are crucial in initiating the body's defenses against various pathogens. Particularly, nucleic acid-sensing receptors, detecting extranuclear DNA or extracellular RNA as DAMP signals, is critical for innate immunity (Desmet and Ishii, 2012; Gong et al., 2020).

Prominent among these RNA sensors are Toll-like receptors (TLRs), RIG-I-like receptors (RLRs), and nucleotide-binding oligomerization domain (NOD)-like receptors (NLRs), which detect foreign viral RNAs to initiate antiviral defenses (Takeuchi and Akira, 2010; Brubaker et al., 2015). Upon recognition of exogenous viral RNAs, these receptors trigger the production of type I interferons (IFNs) and pro-inflammatory cytokines, which are essential elements in the antiviral immune response (Sadler and Williams, 2008). Secreted IFNs, including interferon-α (IFNα) and IFNβ, activate interferon-stimulated genes (ISGs) that control viral infections, establish an antiviral state, and shape the adaptive immune response (Platanias, 2005; Samarajiwa et al., 2009). Type I IFN signaling further amplifies immune responses, facilitating the activation of antigen-presenting cells, the cross-priming of CD8^+^ T cells, and the recruitment of immune cells to combat the invading pathogens (Fuertes et al., 2013; Corrales et al., 2017).

RNA-sensing pathways, essential for antiviral defense, can also interact with endogenous RNAs following dysregulation of cellular processes, contributing to the pathogenesis of various autoimmune and inflammatory diseases (Barrat et al., 2016). Stress-induced alterations in both the quantity and type of endogenous RNAs, along with their abnormal intracellular distribution, may lead to their misidentification as non-self-RNA pathogens, which is termed ‘viral mimicry’ (Roulois et al., 2015). Consequently, host RNAs that are chronically upregulated, mislocalized, or misprocessed, especially during viral infections or under autoimmune conditions, become immunostimulatory (Kristiansen et al., 2011; Wu and Chen, 2014). This inappropriate activation of RNA-sensing pathways by self-ligands can lead to monogenic disorders or complex autoimmune diseases, such as Aicardi–Goutieres syndrome (AGS) and systemic lupus erythematosus (SLE), precipitating the breakdown of self-tolerance.

Despite the aberrant self-RNA sensing in autoimmunity being detrimental, targeting immunogenic endogenous RNAs offers promising avenues for cancer treatment. Radiotherapy, certain chemotherapies, and immunotherapies can activate host RNA-sensing pathways by inducing specific endogenous RNA species, thereby eliciting anti-tumor innate and adaptive immune responses. Elucidating the intricate interplay between immunogenic self-RNAs and cytosolic sensors is imperative, which holds promise for unveiling innovative diagnostics, preventives, and therapeutics of inflammatory diseases driven by the inappropriate recognition of self-RNAs.

## RNA sensors

RNA sensors are activated by exogenous (non-self) viral and microbial RNA, as well as endogenous (self) molecules (Schlee and Hartmann, 2016). The main RNA-sensing PRR families in mammalian cells include TLRs, RLRs, NLRs, 2′,5′-oligoadenylate synthetase (OAS)-like receptors (OLRs), and RNA-dependent protein kinase (PKR). Upon binding to stimulatory RNA, these PRRs trigger a range of immune responses, including antiviral and inflammatory signaling, inhibition of cell growth, and, in certain cases, induction of cell death, to halt viral replication. This section provides an overview of the main RNA sensors and their abilities to recognize endogenous RNAs.

### TLRs

**Figure 1:**
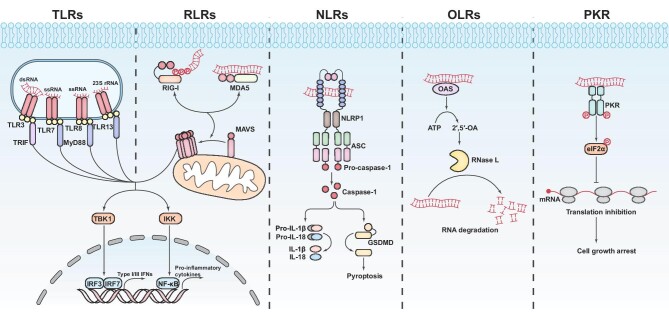
Signaling pathways associated with major RNA sensors, including TLRs, RLRs, NLRs (NLRP1), OLRs (OAS/RNase L), and PKR. RNA-sensing TLRs, predominantly located in the endosomal/lysosomal compartments, include TLR3, TLR7, TLR8, and TLR13. dsRNA in the endosome is detected by TLR3, whereas ssRNA is sensed by TLR7 and TLR8. TLR13, on the other hand, recognizes bacterial 23S rRNA. These receptors signal via MyD88 and TRIF to induce IRF3/IRF7 and type I IFN production and via the transcription factor NF-κB to induce the expression of pro-inflammatory cytokines. The RLR family members, RIG-I and MDA5, localize to the cytoplasm and preferentially recognize 5′ di- or triphosphorylated short dsRNA and long dsRNA, respectively. The activation of RIG-I or MDA5 facilitates aggregation of the adaptor protein MAVS (located in the outer membrane of mitochondria and peroxisomes) via the CARD–CARD interaction, triggering TBK1-dependent IFN production and IKK-dependent pro-inflammatory cytokine expression. The third type of response to RNA is mediated by NLRP1 inflammasome, a macromolecular complex containing the receptor NLRP1, the adaptor ASC, and the effector caspase-1. The mature inflammasome processes pro-caspase-1 into active caspase-1, which then cleaves precursors of inflammatory cytokines (such as IL-1β and IL-18) and the pore-forming protein GSDMD. The GSDMD pore forms in the plasma membrane and induces pyroptosis. The RNA receptor OAS exhibits direct antiviral activity by degrading RNA via the generation of 2′,5′-OA, which serves as a soluble second messenger to activate RNase L. RNase L generates 5′ OH- and 3′ phosphate-containing RNA fragments that can stimulate RIG-I. PKR activation, via dimerization and autophosphorylation, results in the phosphorylation of eIF2α and subsequent inhibition of cap-dependent translation, thus inhibiting cell growth.

TLRs were the first identified and most thoroughly investigated PRR family (Lemaitre et al., 1996). TLR3 and TLR7/8/13 recognize double-stranded RNA (dsRNA) and single-stranded RNA (ssRNA), respectively (Kato et al., 2005; Figure 1). Located primarily in the endosomal/lysosomal compartments, these TLRs are strategically positioned to avoid interaction with self-nucleic acids. Nonetheless, disruptions in this spatial arrangement can lead to autoimmune disease due to the erroneous recognition of self-nucleic acids (Barton and Kagan, 2009).

TLR3, as a dsRNA sensor, plays a critical role in antiviral signaling without RNA sequence specificity, by binding to the ribose phosphate backbone of dsRNA (Karikó et al., 2005; Pohar et al., 2013). The closely related receptors TLR7 and TLR8 primarily recognize ssRNA ligands, leading to a significant induction of pro-inflammatory cytokines in conventional dendritic cells and macrophages, as well as a robust type I IFN response in plasmacytoid dendritic cells (Honda et al., 2005). The ‘orphan’ receptor, TLR13, is expressed in mice and recognizes bacterial 23S ribosomal RNA (rRNA) in a highly sequence-specific manner (Li and Chen, 2012; Oldenburg et al., 2012). Upon ligand binding, TLRs dimerize and recruit adaptor proteins, i.e. myeloid differentiation primary response 88 (MyD88) for TLR7/8/13 and TIR-domain containing protein (TRIF) for TLR3, initiating cascades that ultimately induce IFN and inflammatory mediator transcription (Kawai and Akira, 2010).

### RLRs

Unlike TLRs, RLRs are situated in the cytoplasm of mammalian cells, without any membrane barriers segregating them from the chemical context (Loo and Gale, 2011). Moreover, RIG-I-like helicases (RIG-I) and melanoma differentiation-associated protein 5 (MDA5) are universally expressed, rather than in specialized immune cells (Schlee and Hartmann, 2016; Figure 1). Notably, RIG-I is activated by dsRNAs featuring triphosphate or diphosphate groups at the 5′ end (5′ppp or 5′pp), which are typical characteristics of (sub)genomes and replication intermediates of negative-strand RNA viruses but rarely found in cellular RNAs (typically within a range of 10–19 bp and up to 150–300 bp). In contrast, MDA5 demonstrates a preference for activation by long, irregular dsRNAs (>2000 nt) that are believed to exist in a complex higher-order structure (Hornung et al., 2006; Kato et al., 2006).

Structurally, RLRs feature a conserved helicase core for ATP catalysis and activation, a C-terminal domain (CTD), and two tandem caspase activation and recruitment domains (CARDs) present in RIG-I and MDA5. RIG-I adopts an auto-repressed conformation in the resting state due to interactions between the helicase and CARDs, whereas MDA5 remains an open, flexible conformation without stimulatory RNA. RNA binding to the CTD and helicase of RIG-I and MDA5 induces conformational rearrangement, liberating the CARDs for downstream protein interaction (Kolakofsky et al., 2012). For downstream signal transduction, RIG-I and MDA5 form homotypic contacts with the CARD of their adaptor, mitochondrial anti-viral signaling protein (MAVS, also known as IPS-1, VISA, or Cardiff) (Yoneyama and Fujita, 2010). Upon stimulation, MAVS forms a prion-like filament structure, triggering the formation of a signaling complex that includes TANK-binding kinase 1 (TBK1, also known as IKKε) and the IκB kinase (IKK) kinase complex, composed of IKKα and IKKβ, to activate transcription factors IRF3 and NF-κB, respectively (Wu and Chen, 2014).

### NLRs

NLRs are characterized by the NOD that mediates dNTPase activity and NLR protein (NLRP) assembly. Their activation triggers the production and external release of pro-inflammatory cytokines interleukin 1 (IL-1) and IL-18, stimulates the mitogen-activated protein kinase (MAPK) pathway, and induces apoptosis and pyroptosis (Wen et al., 2013; Figure 1). NLRs are also characterized by a C-terminal Leucine-rich repeat (LRR) domain and an N-terminal effector domain (Kanneganti et al., 2007). The LRR motif array facilitates ligand binding, while the effector domain is involved in connecting with various downstream signaling proteins (Lechtenberg et al., 2014).

NLRP1 is an inflammasome-forming sensor that recognizes microbes to either activate the cytokines IL-1 and IL-18 or stimulate the production of gasdermin D (GSDMD)-mediated pores in the plasma membrane (Martinon et al., 2002). The C-terminal LRR domain and NACHT region of NLRP1 directly sense dsRNAs of >500 bp to activate the inflammasome pathway, relying on both RNA secondary structure and RNA length for sensing (Bauernfried et al., 2021). However, the precise mechanisms of NLRP1 activation remain unclear. NLRP3 is involved in the detection of endogenous RNA DAMPs such as dsRNAs generated by RNA polymerase II (Pol II)-transcribed Alu1 repeats (Tarallo et al., 2012). Details of NLRP3 activation are not fully understood yet, but the process involves an unusual cytoplasmic accumulation of dsRNAs and ssRNAs, along with reactive oxygen species. Activation of NLRP3 prompts itself to unfold and oligomerize, initiating the NLRP3–ASC interaction via Pyr domains. NOD-containing protein 2 (NLRC2, also known as NOD2) has been demonstrated to recognize viral genomic ssRNA during respiratory syncytial virus infection and instigate IRF3-dependent antiviral responses via MAVS (Sabbah et al., 2009). Emerging evidence indicates that certain NLRs, together with specific DEAH-box RNA helicases, can detect viral dsRNA, leading to the activation of type I IFN and/or inflammasome-dependent antiviral responses. For instance, NLRP6 has been identified to play a pivotal role in the intestinal antiviral defense by forming an inflammasome and activating the DHX15–MAVS axis to facilitate IFN production. Specifically, NLRP6 engages with DHX15, establishing a viral sensing complex that recognizes cytosolic long dsRNA and is recruited to MAVS to transcriptionally induce type I and III IFNs as well as ISGs, thereby limiting virus proliferation (Wang et al., 2015). Moreover, virus-derived dsRNA induces NLRP6 liquid–liquid phase separation, positioning it as a key regulator in both inflammasome activation and the type I IFN pathway (Shen et al., 2021). NLRP9b, working with the RNA helicase DHX9, detects short dsRNA and stimulates ASC- and caspase-1-mediated IL-18 synthesis (Zhu et al., 2017).

### OLRs

OASes, which belong to the broader superfamily of nucleotidyltransferases (NTases) and share structural characteristics with proteins such as cyclic GMP–AMP synthase (cGAS), constitute a class of template-independent NTases (Kuchta et al., 2009; Figure 1). In humans, four OAS isoforms are expressed. OAS1, OAS2, and OAS3 exhibit enzymatic activity that facilitates the activation of ribonuclease L (RNase L). In contrast, OASL lacks enzymatic functionality and may play a regulatory role in alternative antiviral immune pathways, one of which mediates the direct binding and activation of RIG-I (Zhu et al., 2014).

OAS1, OAS2, and OAS3 contain one, two, and three tandem repetitions of the NTase domain, respectively. Remarkably, only one NTase domain is catalytically active in OAS2 and OAS3, while the other NTase domains (pseudo-NTase domains) lack the catalytic triad in the active site (Donovan et al., 2015). Intriguingly, OAS3 exhibits a preference for long dsRNAs (>50 bp), although the precise mechanism underlying this specificity remains to be elucidated. Upon dsRNA engagement, the active site of dsRNA-bound OASes undergoes a conformational change, enabling two ATP molecules to be connected via the 2′–5′ linkage. The resultant adenosine 2′–5′-linked oligomers act as second messengers to activate the downstream effector RNase L (Kristiansen et al., 2011).

RNase L is an 80-kDa protein comprising serine/threonine kinase-like and ribonuclease domains and resides as an inactive, self-inhibitory monomer in the cytoplasm. Activated RNase L degrades both cellular and viral ssRNAs with limited sequence specificity, resulting in widespread inhibition of protein synthesis, cellular proliferation, and viral reproduction (Donovan et al., 2017). Notably, RNase L-degraded RNA molecules can activate the cytoplasmic RLR system in a RIG-I-dependent manner, which in turn triggers type I IFN signaling and the pro-inflammatory cytokine response (Malathi et al., 2007).

### PKR

PKR was the first enzyme identified to be linked to the accumulation of intracellular dsRNA and later regarded as one of the RNA sensors (Farrell et al., 1978; Figure 1). It is universally expressed in all types of tissues at basal levels and contains two N-terminal RNA-binding domains, along with a C-terminal serine/threonine protein kinase domain (Dey et al., 2005). The minimal dsRNA length required for PKR activation is reported to be 33 bp, suggesting the necessity of clustering at least two PKR dimers on a single RNA molecule to facilitate interdimeric phosphorylation (Husain et al., 2012).

When PKR binds to dsRNA, it triggers autophosphorylation, dimerization, and the phosphorylation of downstream targets (Dar et al., 2005). The most well-known target of PKR is eukaryotic translation initiation factor 2α (eIF2α). Upon phosphorylation by PKR, eIF2α inhibits the eIF2 complex's ability to initiate mRNA translation in the ribosome, thereby halting protein synthesis and slowing cell growth, which is a program called integrated stress response (ISR) (Mu et al., 2016). In addition to eIF2α phosphorylation, PKR has also been linked to the phosphorylation of IκB, JNK, and MAPK as well as the regulation of apoptosis, autophagy, and metabolic stress (Watanabe et al., 2018).

### Emerging RNA sensors and co-sensors

RNA helicases exhibit growing roles in innate immune detection, in addition to orchestrating RNA metabolism. The SF1 helicase, ZNFX1, localizes to mitochondria, where it binds to viral RNA and promotes type I IFN response in the MAVS-dependent but RLR-independent manner (Wang et al., 2019). Besides RLRs, additional SF2 superfamily members have been demonstrated to detect RNA. Asp–Glu–Ala–Asp (DEAD)-box or Asp–Glu–Ala–His (DEAH)-box helicase proteins are characterized by DEAD or DEAH motifs inside their helicase domains, which also include highly conserved motifs that provide NTP hydrolysis and RNA unwinding capabilities. It has been postulated that DEAD-box or DEAH-box helicases contribute to RNA sensing by either functioning as independent direct RNA sensors, like DDX1 (Zhang et al., 2011), DDX3 (Gringhuis et al., 2017), and DHX33 (Mitoma et al., 2013), or serving as co-sensors to potentiate the activation of RLRs or NLRs, like DHX9 (Zhu et al., 2017), DHX1 (Pattabhi et al., 2019), and DHX29 (Zhu et al., 2018).

Heterogeneous nuclear ribonucleoproteins (hnRNPs), which are typically nuclear or nucleocytoplasmic RNA-binding proteins, are known to control RNA metabolism at the transcriptional or translational level (Geuens et al., 2016). For instance, hnRNPU is proposed to act as a nuclear RNA sensor that interacts with dsRNAs produced by herpes simplex virus 1 in the nucleus, thereby triggering the production of type I IFN (Cao et al., 2019). Additionally, hnRNPU engages in the interaction with SMARCA5 and TOP1, two essential elements of the SWI/SNF nucleosome remodeling complex, to activate enhancers and superenhancers that control the transcription of pro-inflammatory and antiviral genes. In cells lacking MAVS, TBK1, or IRF3, a deficiency in hnRNPU reduces the remaining type I IFN response, indicating that a nuclear signaling pathway directly influences type I IFN transcription.

Z-DNA-binding protein 1 (ZBP1, also known as DAI), which was originally identified as a DNA sensor regulating type I IFN response to synthetic DNA (Takaoka et al., 2007), has been more implicated as an RNA sensor, initiating cell death pathways in response to viral infection. The Zα2 domain of ZBP1 is capable of recognizing either endogenous dsDNA or dsRNA in a ‘Z’ shape (Thapa et al., 2016). For instance, Z-RNA is produced during influenza A virus infection (Zhang et al., 2020), and transcripts of endogenous retroelements, such as Alu, have been identified to carry a Z-RNA ‘signature’. ZBP1, through its Zα2 domain, binds to Z-RNA and activates RIPK3, which can either recruit the RIPK1–FADD–caspase-8 complex to induce apoptosis or engage with mixed lineage kinase domain-like pseudokinase to induce necroptosis.

While it is encouraging to demonstrate that RNA helicases, hnRNPs, and ZBP1 are involved in RNA sensing, further research is required to determine their physiological role in innate immunity and ascertain whether they work by directly detecting RNA or by controlling RNA-dependent signaling through indirect processes. Together, they play a significant role in enabling almost all cells to produce type I IFN in response to viral infection. By triggering the production of multiple ISGs that modify cell metabolism, stop cell cycle, and make cells more susceptible to death, this cell-intrinsic type I IFN activation creates an antiviral state.

## The mechanisms of generating endogenous ligands for RNA sensors

Emerging evidence suggests that in addition to exogenous inducers from virally infected cells, certain forms of endogenous nucleic acids are generated during regular physiological activities or in response to various disturbances. These endogenous RNAs activate the same receptors that have evolved to detect viral RNAs, serving as ‘danger’ signals to alert disruptions in cellular homeostasis. The following section discusses several major endogenous RNA (self-RNA) sources that could potentially lead to constant IFN-I expression.

### Transcription of retrotransposons and production of non-coding RNAs

**Figure 2:**
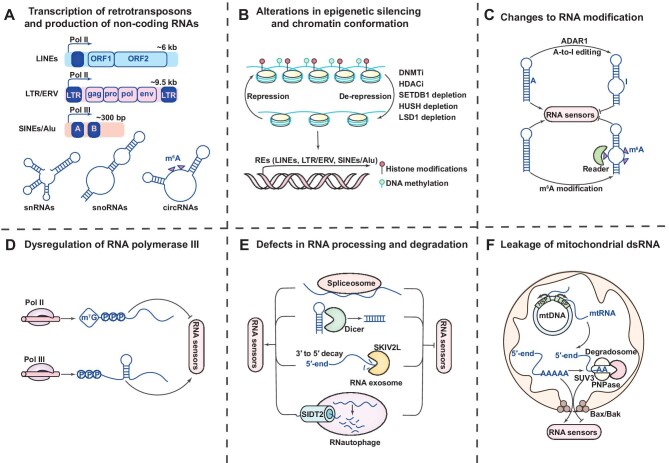
The mechanisms of generating endogenous ligands for RNA sensors. Cells contain a variety of endogenous sources of dsRNA and use a variety of strategies to limit dsRNA synthesis and accumulation. (**A**) General structure of the principal classes of mammalian retrotransposons (LINEs, LTR/ERV, and SINEs/Alu) and non-coding RNAs (snRNAs, snoRNAs, and circRNAs) recognized by RNA sensors. These REs can be translated in both directions or as an inverted repeat, resulting in the formation of foldback hairpin dsRNA or sense-antisense hybrid dsRNA, respectively. Filled rectangles represent transcription regulatory regions, open rectangles represent the main protein-coding regions, and arrows indicate positions of transcription start sites by Pol II or Pol III. A and B represent A-box and B-box in the left monomer of SINEs. (**B**) Chromatin condensation and epigenetic silencing processes often inhibit the biogenesis of RE-based dsRNAs. The aberrant transcriptional activation and reverse transcription of multiple REs are caused by DNMTi, HDACi, or depletion of the histone H3K9 methyltransferase SETDB1, its partner HUSH, or the histone demethylase LSD1. (**C**) Post-transcriptional modifications, such as A-to-I and m^6^A, which both alter dsRNA structure and lessen its immunogenicity, can affect the activity of cellular RNAs. Dysregulation of these (and potentially other) RNA modifications could make normal cellular transcripts appear foreign due to local duplex structure formation. (**D**) Erratic RNA Pol III activation, such as that seen with MYC activation in cancer, can promote the synthesis of dsRNAs with 5′ppp. (**E**) Splicing inhibition can result in an increase in dsRNA levels due to an increase in transcripts with retained introns, which can form double-stranded structures. RNA degradation mechanisms including the RNAase III Dicer, the RNA exosome complex SKIV2L, and the lysosomal RNA transporter SIDT2 may prevent excessive dsRNA buildup. (**F**) Due to their bacterial heritage, mitochondria possess circular mtDNA, which is bidirectionally transcribed from promoters on each strand and may produce large quantities of double-stranded mtRNA. The mtRNA degradosome, which contains the helicase SUV3 and PNPase, typically regulates mitochondrial dsRNA levels. When the mtRNA degradosome is inhibited, mtRNA is released via inner mitochondrial membrane herniation through Bax/Bak pores.

A significant portion of RNA molecules that accumulate in the cytoplasm and bind to various RNA sensors during stress originates from retrotransposons and repetitive non-coding RNAs. Retrotransposons, which include both truncated and full-length internal retrotransposable elements (REs), constitute ∼40% of human and mouse genomes. These elements propagate within the host genome through a ‘copy and paste’ mechanism (Fabre et al., 2012). The highly repetitive nature of these REs leads to an accumulation of dsRNAs, detectable within complexes involving diverse cytoplasmic RNA sensors long interspersed nuclear elements (LINEs), long terminal repeats (LTRs), endogenous retroviral elements (ERVs), and short interspersed nuclear elements (SINEs, such as Alu1 repeats in humans and B1/B2 repeats in mice) (Boelens et al., 2014; Guler et al., 2017; Chiang et al., 2018; Figure 2A).

LINEs, which account for 20% of the human genome, are endogenous REs that possess active genes for reverse transcription and genome integration (Kassiotis and Stoye, 2016). They are transcribed by Pol II from internal promoters in the 5′ and 3′ UTRs, which are associated with the activation of RIG-I-dependent IFN signaling (Guler et al., 2017). LTR retroelements, which make up ∼8% of the human genome, represent another category of endogenous REs, flanked by LTRs at their ends (Wicker et al., 2007). ERVs, also classified as LTR retroelements, can encode envelope proteins for viral particle formation (Vargiu et al., 2016).

Notably, LTR sequences have been found to be enriched in RIG-I-activating exosomes (Boelens et al., 2014). SINEs, particularly Alu elements in humans, are well-known REs that can be triggered by genotoxic stress and activate various RNA sensors. Characteristically, SINEs are AT-rich repetitive sequences of ∼300 bp long, capable of integrating into the genome, and frequently form tracts of repeating elements in either direct or reverse orientation, comprising ∼10% of the human genome. Alu elements exhibit a preference for the integration into intergenic or intronic regions of protein-coding genes. They can be transcribed by RNA Pol II as part of lengthy mRNAs for protein-coding genes or by RNA Pol III as single short RNAs (Ablasser et al., 2009). Notably, the editing enzyme adenosine deaminase acting on RNA 1 (ADAR1) post-transcriptionally modifies Alu:Alu hybrids, converting adenosine (A) to inosine (I) and causing structural changes in the dsRNA due to mismatches and bulges within the duplex (Ahmad et al., 2018). These modified hybrids are not recognized by the MDA5 sensor.

Small nuclear RNAs (snRNAs) and small nucleolar RNAs (snoRNAs) represent important types of small non-coding RNAs (Figure 2A). SnRNAs, part of the nucleoplasmic spliceosome (Chari and Fischer, 2010), can function as DAMPs recognized by RLRs and TLRs (Bernard et al., 2012), while snoRNAs are involved in rRNA modification (Falaleeva et al., 2017) and serve as PKR ligands (Youssef et al., 2015). Circular RNAs (circRNAs), generated by back-splicing of pre-mRNAs, have unique secondary structures and RNA modification patterns, setting them apart from linear RNAs (Chen, 2016) and leading to different interactions with nucleic acid sensors and immune receptors. Notably, the pronounced dsRNA structure of circRNAs enhances PKR binding (Liu et al., 2019), while the N6-methyladenosine (m^6^A) modification on these circRNAs can prevent RIG-I detection (Chen et al., 2019).

### Alterations in epigenetic silencing and chromatin conformation

Multiple regulatory mechanisms are utilized to silence integrated REs, including epigenetic modifications of DNA and chromatin, condensation of chromatin domains into heterochromatin structures, and recruitment of transcriptional suppressors to promoter regions (Thompson et al., 2016). The inactivation of these silencing mechanisms leads to RNA production by repetitive RE genes and the activation of the innate immune response through binding to RNA sensors. DNA methylation, a critical player in transcriptional regulation, targets endogenous promoters in various REs, including LINEs, LTR/ERV, and SINEs (Goodier, 2016). This indicates that inhibition of DNA methylation could trigger the transcriptional activation of REs, consequently leading to the formation of abnormal dsRNAs and their interactions with RNA sensors like MDA5, TLR3, and OASes (Chiappinelli et al., 2015; Roulois et al., 2015; Figure 2B).

Numerous studies have shown that 5-aza-2′-deoxycytidine (5-azacitidine), a DNA methyltransferase inhibitor (DNMTi) and common chemotherapeutic drug, significantly induces the transcriptional upregulation of epigenetically repressed REs (Mehdipour et al., 2020). The action of such chemicals typically increases the expression of endogenous retroviruses, which are crucial for MDA5 activation. Similar to DNMT inhibition, various histone modifications associated with repressed heterochromatin regions of chromatin are linked to REs, contributing to their transcriptional silencing (Goodier, 2016). For instance, trimethylation of histone H3K9, an epigenetic marker, is associated with transcriptional repression in REs and heterochromatin regions. Depleting the H3K9 methyltransferase SETDB1 or its partner, human silencing hub (HUSH), induces a wide array of REs, leading to subsequent activation of RLR signaling (Cuellar et al., 2017; Tunbak et al., 2020). Similarly, inhibiting histone demethylase LSD1 (also known as KDM1A) activates TLR3 and RLRs (Sheng et al., 2018). Thus, epigenetic inhibitors, such as DNMTi and histone deacetylase inhibitor (HDACi), play crucial roles in controlling endogenous dsRNA synthesis, especially those originating from REs. The dynamic interaction between epigenetic modifiers and the innate immune system opens a promising area of exploration in the field of cellular biology.

### Changes to RNA modification

RNA modifications have been found to significantly impact RNA metabolism, secondary structure, and protein interactions. Defects in the implementation or detection of RNA modifications can result in self-RNAs appearing as foreign, thereby initiating immune responses. Two major forms of RNA modifications, adenosine deamination and methylation, alter local dsRNA structures and subsequently decrease the recognition of endogenous RNAs by dsRNA sensors (Figure 2C).

A-to-I RNA editing is a common post-transcriptional RNA modification crucial for mammalian survival (Nishikura, 2010). ADAR1, a dsRNA-specific adenosine deaminase with low sequence dependence, binds to dsRNA to facilitate this A-to-I editing, which transforms stable A–U pairs into less stable I–U pairs, thus disrupting the dsRNA structure primarily generated by inverted repetitive sequences, such as SINEs (Levanon et al., 2004; Ramaswami et al., 2012) and preventing dsRNA recognition by MDA5, PKR, OASes, and ZBP1 (Liddicoat et al., 2015; Li et al., 2017; Chung et al., 2018; Jiao et al., 2022). Furthermore, the cytoplasmic ADAR1 isoform p150 can suppress PKR activation through dsRNA-binding competition (Hu et al., 2023).

m^6^A is another important modification on RNA. The absence of m^6^A modification induces dsRNA synthesis and dsRNA sensor activation (Roundtree et al., 2017). Most protein-coding RNAs feature m^6^A modification in their native state. On these RNAs, m^6^A modification serves as a structural switch, preventing base pairing and the formation of dsRNAs (Liu et al., 2015). m^6^A modification can also influence the expression of REs, although the effects vary across species and cell types (Gao et al., 2020). RIG-I is the principal cytosolic receptor that detects viral dsRNA in an m^6^A-dependent manner, while MDA5 plays a lesser role.

Aside from A-to-I editing and m^6^A, >170 additional RNA modifications have been identified, though their roles remain largely unknown (Boccaletto et al., 2018). Some of these RNA modifications have the potential to influence dsRNA synthesis and/or immune receptor recognition. Pseudouridine, 2-thiouridine, or 5-methylcytidine incorporation into exogenously inserted RNAs (like therapeutic mRNA) enables them to evade detection by TLRs, RIG-I, and PKR (Anderson et al., 2010). Conversely, 5-methyluridine activates PKR, indicating that immune receptors respond differently to various RNA modifications.

### Dysregulation of RNA Pol III

Unlike RNA Pol II transcripts, which are processed to have a 7-methylguanosine (m^7^G) cap at the 5′ end, many RNA Pol III transcripts, such as U6 snRNA, 5S rRNA, 7SK RNA, and 7SL RNA, retain 5′ppp in nascent transcripts, although a few acquire a monomethyl group on the γ-phosphate of 5′ppp (Dieci et al., 2013). Many Pol III transcripts also contain RNA secondary structures, which together with 5′ppp, confer on them the potential to stimulate RIG-I. For example, 7SL RNA is a component of the signal recognition particle (SRP) ribonucleoprotein complex that interacts with the ribosome and sends nascent proteins to the endoplasmic reticulum for secretion or membrane insertion (Nabet et al., 2017). Like non-self-nucleic acids, 7SL RNA possesses a 5′ppp signature with secondary structures, which could stimulate RIG-I under certain conditions (Bowie and Fitzgerald, 2007). Under normal physiological conditions, 7SL RNA is shielded from RIG-I detection due to its association with SRP protein partners (Figure 2D).

### Defects in RNA processing and degradation

Several lines of evidence suggest that disruptions to RNA processing and degradation pathways could result in the accumulation of immunostimulatory dsRNAs within cells. Similar to the action of DNMTi in stimulating ERV expression, small compounds that disrupt spliceosome function, especially in combination with hyperactivation of MYC, may result in cytoplasmic dsRNA accumulation and trigger antiviral-like responses (Bowling et al., 2021). The inhibition of spliceosomal activity leads to an increase in intron-retained transcripts, potentially giving rise to dsRNA structures (Blango and Bass, 2016; Figure 2E).

While most cellular ribonucleases are ineffective in degrading dsRNAs, Dicer possesses an RNase III domain that specializes in cleaving dsRNAs. Known primarily for processing microRNA precursors to generate mature microRNAs, Dicer also processes long dsRNAs from both internal and external sources, creating small interfering RNAs (Watanabe et al., 2008). Many of these endogenously derived small interfering RNAs originate from sense–antisense hybrids of REs or pseudogenes and transcripts containing inverted repeats of retroelements (Yang and Kazazian, 2006). Consequently, any disturbances in Dicer functionality can lead to an accumulation of retroelement transcripts (Heras et al., 2013).

SKIV2L, an essential RNA helicase within the RNA exosome, plays a pivotal role in cytosolic RNA turnover (Halbach et al., 2013). Its helicase activity assists the exosome in unraveling RNA secondary structures, a critical step for the removal of damaged RNA and mRNA turnover (Garneau et al., 2007). Deficiencies in SKIV2L can lead to RIG-I hyperactivation, while the identity of the RIG-I-stimulatory RNAs and the mechanism of RIG-I activation remain unclear (Eckard et al., 2014). In addition to exosome-mediated degradation, cytosolic RNAs can also be removed via ‘RNautophagy’, a process that RNAs are directly engulfed by lysosomes through an RNA transporter (Aizawa et al., 2016). Given that SIDT2 acts as a bidirectional dsRNA transporter that is an orthologue of SID-1 (Nguyen et al., 2017), it would be intriguing to investigate whether the impaired SIDT2 function contributes to the accumulation of endogenous dsRNAs.

### Leakage of mitochondrial dsRNA

Mitochondrial DNA (mtDNA), typically present in thousands of copies per cell, yields nucleic acid species with immunostimulatory potential during its transcription and replication, such as dsRNAs, uncapped mRNAs, and RNA–DNA hybrids (Fernández-Silva et al., 2003). Recently, mitochondrial RNA (mtRNA) has been recognized as a potential ‘danger’ signal indicative of cellular dysfunction, thereby eliciting responses from the innate immune system (Grazioli and Pugin, 2018; Figure 2F).

The mitochondrial degradosome, comprising the ribonuclease polynucleotide phosphorylase (PNPase) and the RNA helicase SUV3, is required to prevent dsRNA formation during the bidirectional transcription of the mitochondrial circular genome (Dhir et al., 2018). SUV3 depletion leads to mitochondrial dsRNA accumulation, whereas PNPase depletion results in dsRNA accumulation in both mitochondria and cytoplasm. MDA5 detects mtRNA in the cytosol, possibly entering through BAX–BAK pores in PNPase-depleted cells and triggering a type I IFN response (Dhir et al., 2018). Additionally, loss-of-function (LOF) mutation in N-glycanase (NGLY1), a conserved deglycosylation enzyme, leads to sustained activation of cytosolic nucleic acid-sensing pathways, potentially due in part to mtRNA and mtDNA (Yang et al., 2018). The precise relationship between NGLY1, mitochondrial homeostasis, and type I IFN signaling remains unclear but may involve mitophagy and/or proteasome activity via the transcription factor NRF1 (Lam et al., 2017).

Hypomorphic mutations in RNASET2 and TRNT1 have also been linked to the upregulated type I IFN signaling (Tonduti et al., 2016). RNASET2 potentially contributes to the degradation of mtRNAs within the intermembrane space (Liu et al., 2017). TRNT1 plays a crucial role in tRNA aminoacylation by adding CCA residues to the 3′ end of mitochondrial and cytosolic tRNA molecules, and disease-causing mutations in this gene result in faulty mitochondrial translation (Sasarman et al., 2015). Although these findings are preliminary, they suggest a connection between IFN signaling and the reduced RNASET2 activity or TRNT1 dysfunction, which warrants further exploration. It has also been proposed that mitochondrial dsRNA can mediate the innate immune response following ionizing radiation exposure. Both nuclear DNA and mtDNA are susceptible to the damage caused by ionizing radiation, which can lead to their leakage into the cytoplasm as a result of compromised mitochondrial membrane integrity.

Moreover, a recent study has revealed that the leakage of mtRNA occurs when the tricarboxylic acid cycle enzyme fumarate hydratase (FH) is suppressed following prolonged lipopolysaccharide stimulation. The leaked mtRNA subsequently activates RNA sensors TLR7, RIG-I, and MDA5, resulting in enhanced production of IFNβ (Hooftman et al., 2023).

Overall, dsRNAs transcribed from various sources, including LINEs, LTR/ERV, and SINEs/Alu, as well as snRNAs and circRNAs, are well-documented cell-intrinsic ligands (RNA DAMPs) for RNA sensors. These ligands can be activated by a variety of stressors, including irradiation, chemotherapy, kinase inhibitors, and epigenetic modifiers, leading to the accumulation of abnormal RNA transcripts. Such dsRNA accumulation is positively regulated by Pol II/Pol III transcription and cell cycle-dependent nuclear cellular proteins and negatively regulated by the adenosine deaminase ADAR1, the RNAase III Dicer, the cytoplasmic RNA-degrading complex SKIV2L, and RNase L. The interaction between these RNA ligands and cytoplasmic RNA sensors, including TLR3/7/8, RLRs, NLRP3, and PKR, triggers downstream signaling that result in three key outcomes: type I IFN signaling activation, inflammatory response activation, and translational shutdown/cell death activation in cell subpopulations with imbalanced RNA stress (Figure 2).

## Diseases caused by sterile activation of RNA sensors

Cellular RNA sensors have the finely tuned activation thresholds, enabling them to tolerate specific levels and types of dsRNA (Chen and Hur, 2022). Normally, cellular dsRNA levels remain well below the activation threshold of these sensors. However, during RNA virus infections or excessive accumulation of immunogenic endogenous dsRNA, dsRNA levels can exceed the threshold, leading to sensor activation. Dysregulated dsRNA-sensing pathways, characterized by heightened sensor sensitivity, ligand-independent activation, or impaired negative regulation of type I IFN signaling, can further lower the activation threshold. Together, these contribute to inappropriate type I IFN expression, potentially resulting in systemic autoinflammation and various autoimmune manifestations. Notable examples of type I IFN diseases encompass monogenic autoinflammatory disorders like AGS and Singleton–Merten syndrome (SMS), as well as multifactorial systemic autoimmune diseases such as SLE.

AGS was initially characterized in the 1980s as a hereditary encephalopathy and is known for elevated type I IFN levels that resemble symptoms of congenital viral infections (Aicardi and Goutières, 1984). So far, mutations in nine different genes (*AGS1*–*AGS9*) have been identified as causative factors for AGS (Crow and Stetson, 2022). Among these, mutations in *ADAR* (*AGS6*) and *IFIH1* (*AGS7*) generate aberrant signals that activate the RNA-sensing pathway and cause AGS. ADAR, an adenosine deaminase, edits dsRNA by converting adenosine bases to inosines (Liddicoat et al., 2015). Mutations in ADAR result in the accumulation of immunogenic dsRNA and the increased MAVS-dependent type I IFN production (Mannion et al., 2014). In contrast, gain-of-function (GOF) mutations in *IFIH1* lead to direct MAVS-dependent IFN production with variable disease severity. SMS is characterized by symptoms such as vascular calcifications, periodontal disease, alveolar bone loss, skeletal deformities, and osteoporosis and caused by heterozygous GOF mutations in *IFIH1* or *DDX58*, which encodes RIG-I (Jang et al., 2015).

SLE is a chronic autoimmune disease characterized by symptoms including fevers, fatigue, dermatitis, and arthritis (Rahman and Isenberg, 2008). SLE is a multifactorial systemic disease influenced by both genetic and environmental factors (Harley et al., 2009). The loss of self-tolerance contributes to the development of autoantibodies, including hose against RNA-binding proteins and dsRNA (Kirou et al., 2005), leading to tissue inflammation through immune complex deposition. Notably, patients with SLE exhibit a significant IFN signature in peripheral blood cells, underscoring the role of type I IFN in the pathogenesis of SLE (Baechler et al., 2003).

Subsequently, we will focus on the pathogenic roles of inappropriate activation of RNA sensors and their underlying mechanisms.

### Hyperactivation of RNA sensors

#### RLRs

GOF mutations in the RLR members *IFIH1* (encoding MDA5) and *DDX58* (encoding RIG-I) have been associated with several rare, inherited autoimmune diseases, including classical and atypical SMS, certain subtypes of AGS, and SLE, all characterized by elevated levels of IFN-I (Bursztejn et al., 2015; Jang et al., 2015; Rice et al., 2020; Table 1). *DDX58* variants (c.1529A>T [p.E510V], c.803G>T [p.C268F], c1118A>C [p.E373A], and c1551G>C [p.Q517H]) have been reported to correlate with the increased IFN levels and susceptibility to atypical (milder) and classic (severe) SMS defects, presenting with dental dysplasia, glaucoma, psoriasiform skin rash, aortic calcifications, and skeletal dysplasia (Jang et al., 2015; Ferreira et al., 2019; Prasov et al., 2022). Among them, E373A and C268F mutations are located in the SF2 ATP-binding and hydrolysis motif I and motif II of RIG-I, respectively. They are thought to impair ATPase activity without any effect on ATP binding and form long-lived ATP-bound complexes on stem RNAs to increase interaction with self-RNA, leading to abnormal innate immune signaling (Devarkar et al., 2018).

**Table 1:** Mutations in RNA-sensing pathway linked to autoimmunity.

**Gene (alternative name)**	**Function**	**Mutations**	**Domains**	**Mutation effect**	**Phenotype**	**References**
*DDX58* (RIG-I)	dsRNA sensor	C268F, E373A	Hel1 domain	GOF (AD)	SMS	Jang et al. (2015); Ferreira et al. (2019)
		E510V, Q517H	Hel2i domain	GOF (AD)	SMS	Ferreira et al. (2019); Prasov et al. (2022)
		R109C	CARD2 domain	GOF (AD)	Lupus nephritis	Peng et al. (2023)
*IFIHI* (MDA5)	dsRNA sensor	R822Q	Hel2 domain	GOF (AD)	SMS	Rutsch et al. (2015)
		R337G, R779C, G495R, D393V, R720Q, A452T, L372F	Helicase domain	GOF (AD)	AGS	Oda et al. (2014)
		A489T	Hel1 domain	GOF (AD)	AGS, AGS	Oda et al. (2014); Bursztejn et al. (2015)
		G821S, A946T	Hel2 domain and CTD	GOF (AD)	SLE	Funabiki et al. (2014); Van Eyck et al. (2015)
		R779H	Pincer domain	GOF (AD)	AGS, SLE	Rice et al. (2014); Van Eyck et al. (2015)
*TLR7*	ssRNA sensor	rs3853839	3′ UTR	GOF (XR)	SLE	Kawasaki et al. (2011); Wang et al. (2014)
		Y264H	LRR domain	GOF (XD)	SLE	Brown et al. (2022)
*TLR8*	ssRNA sensor	P432L	LRR domain	GOF (XD)	IEL	Aluri et al. (2021)
		F494L, G527D	/	GOF (XD)		
*NLRP1*	dsRNA sensor	R726W	The linker region between the NACHT and LRR domains	GOF (AD)	Vitiligo and rheumatoid arthritis	Yu et al. (2018)
*PKR*	dsRNA sensor	N32T, G130R	dsRNA-binding domain	GOF (AD, AR)	Dystonia	Kuipers et al. (2021); Magrinelli et al. (2022)
*ADAR1*	Adenosine deaminase (RNA editing)	R892H, K999N, G1007R, Y1112F, D1113H	Adenosine deaminase domain	LOF (AR)	AGS	Rice et al. (2012)
		A870T, I872T	The linker region			
		P193A	Z-DNA/Z-RNA-binding domain			
*SKIV2L*	RNA helicase (the RNA exosome complex)	V341G	ATP-binding domain	LOF (AR)	THES	Fabre et al. (2012)
*PNPT1* (PNPase)	Polynucleotide (mitochondrial degradosome)	S70P, D713Y	/	LOF (AR)	COXPD	Dhir et al. (2018)
*NGLY1* (PNGase)	*N*-deglycosylation (UPR)	R401X	/	LOF (AR)	CDDG	Lam et al. (2017)

AR, autosomal recessive; AD, autosomal dominant; XR, X chromosome recessive; XD, X chromosome dominant; COXPD, combined oxidative phosphorylation deficiency; CDDG, congenital disorder of deglycosylation.

In addition, the E510V and Q517H mutations are located on the same α-helix of helicase 2 insertion (Hel2i) domain and cause a loosened latch-gate engagement in apo RIG-I, which, in turn, gradually dampens its self-RNA proofreading ability, leading to increased immunopathy (Lei et al., 2022). The known endogenous RIG-I ligands include 3′ UTR of *Nf-κb1* mRNA (Zhang et al., 2013), cleavage product RNA of RNase L (Malathi et al., 2007), 28S rRNA expansion segment 7 L (Lassig et al., 2016), and transcript of 5S rRNA pseudogene (Chiang et al., 2018). Further research into the physiological relevance of these RNAs in autoimmunity is needed. Moreover, a new *DDX58* pathogenic variant (c.325C>T; p.Arg109Cys), potentially leading to RIG-I hyperactivation without ligands, has been identified in lupus nephritis patients (Peng et al., 2023). The R109 residue is located in the interface between CARD2 domain and DEAD helicase domain, while the R109C mutation attenuates the interaction between CARDs and DEAD helicase domain, causing the exposure and release of CARDs from autoinhibition state, which results in RIG-I signaling activation and type I IFN signaling upregulation (Peng et al., 2023).

In the case of MDA5, a missense mutation in the *IFIH1* gene (c.2465G>A; p.Arg822Gln) has been identified as the cause of classical SMS, characterized by dental anomalies, aortic and valvular calcification, glaucoma, and an enhanced type I IFN signature gene expression pattern (Rutsch et al., 2015). This mutation is located in the Hel2 domain of MDA5 and induces conformational changes that promote the formation and stability of MDA5 filaments on dsRNA, resulting in constitutive sensor activation. Additional AGS-associated missense mutations in *IFIH1* (p.Arg337Gly, p.Arg779Cys, p.Gly495Arg, p.Asp393Val, p.Arg720Gln, p.Arg779His, p.Ala452Thr, and p.Leu372Phe) are all positioned close to the RNA-binding or ATP-binding sites in the MDA5 helicase domain (Oda et al., 2014). Among these mutations, G495R was found to tolerate A-to-I modification, thus allowing the stimulation by Alu:Alu hybrids, which are abundant in the cytosol (Ahmad et al., 2018). Moreover, a distinct mutation in *IFIH1* (p.Ala489Thr) has overlapping features of AGS and SMS and is associated with upregulated ISGs, suggesting a potential phenotypic overlap in various diseases (Bursztejn et al., 2015).

In SLE, multiple variants in the *IFIH1* gene (p.Gly821Ser, p.Ala946Thr, and p.Arg779His) have been identified (Van Eyck et al., 2015), and recombinant MDA5 variants G821S and A946T exhibit the altered conformations compared to the wild-type, potentially activating downstream signaling in the absence of RNA (Funabiki et al., 2014). These ‘hyperactive’ MDA5 variants correlate with the elevated type I IFN levels in SLE patients. Interestingly, some SLE patients carrying an AGS-associated MDA5 variant (p.Arg779His) present with early-onset SLE without AGS symptoms, indicating diverse clinical manifestations caused by *IFIH1* mutations (Rice et al., 2014).

Mechanistically, these GOF mutations may enhance RNA-binding and misrecognition of endogenous RNAs, leading to self-triggered signaling (Rice et al., 2014), or activate the RLR–MAVS signaling pathway in a ligand-independent manner by releasing the auto-repression of the two CARDs (Funabiki et al., 2014). Notably, there is a higher prevalence of pathogenic mutations in MDA5 than in RIG-I, which may be attributed to structural and ligand specificity differences between the two proteins.

#### TLRs

Multiple TLRs are associated with SLE and its clinical manifestations, including the production of autoantibodies and type I IFN. A 2-fold increase in *Tlr7* expression promotes autoimmunity in a lupus-prone mouse strain, while its higher expression levels induce a lupus-like autoimmune disease in a typically healthy mouse strain (Deane et al., 2007). Both induced and spontaneous SLE are resistant in *Tlr7*-deficient mice (Christensen et al., 2006; Table 1). Notably, SLE patients from Eastern Asian countries have been reported to carry single nucleotide polymorphisms associated with increased *TLR7* expression (Kawasaki et al., 2011). Particularly, the TLR7 rs3853839-G>C variant was identified in a Taiwanese population to increase the risk for SLE (Wang et al., 2014). In addition, a missense TLR7^Y264H^ variant has been identified as a GOF mutation that exhibits higher sensitivity to guanosine and 2′,3′-cGMP in human lupus (Brown et al., 2022). The genetic information of TLR8 seems more complex. TLR8 deficiency in mice results in lupus-like conditions, presumably mediated by the increased TLR7 expression (Demaria et al., 2010). By contrast, transgenic expression of human TLR8 in mice causes an increased cytokine production with hyperinflammation (Guiducci et al., 2013). A recent study found that mosaic and germline GOF variants in *TLR8* lead to novel inborn errors of immunity with lymphoproliferation, neutropenia, infectious susceptibility, B- and T-cell defects, and in some cases, bone marrow failure (Aluri et al., 2021). These *TLR8* variants induce hypersensitivity to ligand stimulation in patient-derived cells, leading to heightened NF-κB activation and increased cytokine production (Aluri et al., 2021; Table 1). Unlike human TLR8, which responds to ssRNA, mouse TLR8 appears to be nonfunctional on its own. There may be yet-to-be-identified differences in ligand specificity between human and mouse TLR8. So far, emerging evidence has suggested that TLR7 and TLR8 are capable of spontaneous activation with potential pathogenic implications in humans.

#### NLRs

NLRP1 mutations in both coding and non-coding regions are implicated in various diseases, including metabolic disorders, cancer, and autoimmune diseases (Drutman et al., 2019). Specific polymorphisms in the *NLRP1* gene leading to its constitutive activation are associated with an elevated risk of autoimmune disorders such as vitiligo and rheumatoid arthritis (Yu et al., 2018). In humans, missense mutations inherited in a recessive manner are found in the linker region between the NACHT and LRR domains of NLRP1. One such mutation, p.Arg726Trp, causes NLRP1-associated autoinflammation with arthritis and dyskeratosis (NAIAD) and is linked to the increased caspase-1 activity (Grandemange et al., 2017; Table 1). However, the potential involvement of endogenous dsRNA in these situations remains uncertain, as NLRP1 can be activated by other PAMPs besides dsRNAs. Further investigation is needed to elucidate the significance of endogenous dsRNA in these contexts.

#### PKR

Irregular PKR activation has been implicated in various diseases, including dystonia, SLE, Alzheimer's disease, and Huntington's disease (Liu et al., 2019; Lee et al., 2020; Kuipers et al., 2021; Reimer et al., 2021). Dystonia, in particular, offers compelling genetic evidence implicating PKR in disease onset (Table 1). Specific mutations in genes encoding PKR and its activator, PACT, have been identified in certain dystonia patients. These mutations result in the sustained activation of PKR and subsequent initiation of the ISR (Burnett et al., 2020; Kuipers et al., 2021).

### Excessive accumulation of immunogenic endogenous RNAs

Sterile immune activation can occur regardless of changes in dsRNA sensors but be driven by alterations in endogenous RNAs that arise from modifications or localized accumulation of RNAs.

#### Aberrant post-transcriptional modification of RNAs

ADAR1-mediated adenosine deamination is a key RNA modification implicated in autoimmune diseases, such as AGS (Rice et al., 2012). ADAR1 has two isoforms: the nuclear-localized p110 and the predominantly cytoplasmic p150, the latter being induced by IFN. The p150 isoform comprises a deaminase domain, dsRNA-binding domain, Zβ domain, and Z-nucleic acid-binding Zα domain. LOS mutations in ADAR1 can lead to dysregulated IFN signaling, contributing to disease pathogenesis (Rice et al., 2012). Complete deficiency of Adar1 or its p150 isoform in mice results in the elevated ISG levels in embryos and mortality during embryonic days 11.5–12.5, potentially explaining the absence of AGS patients with *ADAR1*^−/−^ or *ADAR1*^p150−/p150−^ mutations (Hartner et al., 2004; Wang et al., 2004; Ward et al., 2011). However, concurrent deletion of MDA5 or MAVS in mice extends survival to only ∼2 days after birth, indicating that the embryonic lethality is mainly caused by MDA5–MAVS-induced type I IFN signaling, whereas the early postnatal death may be mitigated by other mechanisms (Hartner et al., 2004; Wang et al., 2004; Ward et al., 2011). AGS-related ADAR1 mutations comprise nonsense, frameshift, and missense mutations distributed across the catalytic, Zα, and RNA-binding domains, impairing the functions of these domains to varying degrees. A mouse model with bi-allelic E861A mutations, completely abolishing A-to-I editing activity, phenocopies the embryonic lethality of *Adar1*^−/−^ mice and develops a spontaneous MDA5-mediated type I IFN response, underscoring the critical role of A-to-I editing in averting immunopathology (Liddicoat et al., 2015). However, the lethality of *Adar1*^E861A/E861A^ mice can be completely rescued by simultaneous knockout of MDA5 (Liddicoat et al., 2015), different from that of *Adar1*^−/−^ mice, indicating that the suppression of MDA5 activation prevents embryonic lethality mainly through editing-dependent mechanisms, while ADAR1 also has editing-independent functions. Notably, over half of heterozygous patients carry mutations in the Zα domain, like P193A, alongside mutations impairing the editing activity of ADAR1, such as R892H, or null allele (Rice et al., 2012; Song et al., 2022). Interestingly, *Adar1*^P195A/P195A^ mice appear indistinguishable from wild-type controls and exhibit a mild upregulation of ISGs, while *Adar1*^P195A/−^ or *Adar1*^P195A/p150−^ mice exhibit pathologies, aberrant type I IFN expression, and mortality (median survival, 21 days or 40 days, respectively), indicating that the P195A mutation alone is not pathogenic but p150 isoform may play a role in preventing immunopathology (Maurano et al., 2021). Further genetic and biochemical analyses found that p150 isoform can provide a negative feedback through A-to-I editing of cytosolic dsRNA and sequestration of dsRNA or Z-RNA (Maurano et al., 2021; Nakahama et al., 2021; Tang et al., 2021; De Reuver et al., 2022; Jiao et al., 2022; Hu et al., 2023). In the most common human ADAR-AGS mutation, hemizygous expression of the P195A Zα domain-mutant ADAR1-p150 fails to adequately edit and sequester endogenous dsRNA, leading to uncontrolled MDA5 activation. This triggers a pathological MDA5–type I IFN-mediated positive-feedback loop, upregulating PKR and ZBP1 expression. Furthermore, the insufficient buffering effect of the impaired mutant p150 allows for PKR and ZBP1 activation by the accumulated dsRNA or Z-RNA, inducing the ISR and cell death, respectively, thereby contributing to immunopathology (Maurano et al., 2021; Nakahama et al., 2021; Tang et al., 2021; De Reuver et al., 2022; Jiao et al., 2022; Hu et al., 2023).

#### Augmented intracellular accumulation of RNAs

Enhanced cytosolic RNA concentrations may arise from the increased production or impaired degradation of these ligands. Leaked mtRNAs contribute to this enhancement, thereby triggering the production of IFN and ISGs. Intriguingly, fibroblasts derived from patients with bi-allelic pathogenic *PNPT1* variants showed an accumulation of mitochondrial unprocessed *PNPT1* transcripts, while peripheral blood demonstrated an increased IFN response (Dhir et al., 2018; Table 1). Furthermore, *NGLY1*-deficient patient cells exhibited more fragmented mitochondria and elevated expression of several ISG transcripts compared with the health control (Yang et al., 2018). Additionally, macrophage FH is known to inhibit mtRNA-mediated IFN production; thus, the reduced FH expression in SLE patient-derived cells suggests a potential pathogenic role for leaked mtRNA in SLE (Hooftman et al., 2023).

RNA degradation plays a pivotal role in preventing the accumulation of immunogenic aberrant RNA transcripts and metabolic byproducts. The cytosolic RNA-degrading machinery, known as the exosome, includes the component SKIV2L, which limits the activation of RLR signaling (Eckard et al., 2014). Bi-allelic mutations in human *SKIV2L* gene give rise to trichohepatoenteric syndrome (THES), a rare disorder characterized by growth retardation, craniofacial abnormalities, and intestinal dysfunction (Fabre et al., 2012). While most of these symptoms are likely attributed to the involvement of SKIV2L in RNA turnover, it is noteworthy that SKIV2L-THES patients (c.1635insA) exhibit a peripheral blood IFN signature similar to that seen in AGS patients (Eckard et al., 2014). Collectively, these studies indicate that genetic or environmental disturbances affecting the regulatory processes can disrupt the balance between RNA sensors and endogenous ligands, initiating cascades of signaling events that ultimately lead to self-mediated sterile inflammation and inflammatory disorders.

## Therapeutic applications

The involvement of endogenous dsRNA in the aberrant activation of RNA-sensing pathways and subsequent development of inherited autoimmune diseases has sparked significant interest in the development of compounds targeting these processes for potential therapeutic interventions. Furthermore, acute induction of immunostimulatory endogenous dsRNAs holds great promise in cancer therapy (Table 2).

**Table 2:** Autoimmune diseases targeting clinical trials.

**Disease**	**Trial compounds**	**Target**	**CT identifiers**
AGS	Abacavir, lamivudine, and zidovudine	Reverse transcriptase inhibitors	NCT04731103
AGS	Tenofovir and emtricitabine	Reverse transcriptase inhibitors	NCT03304717
SLE & CLE	M5049 (Enpatoran)	TLR7/8 inhibitor	NCT05162586
SLE	BMS-986256 (Afimetoran)	TLR7/8 inhibitor	NCT04269356 and NCT04895696
Plaque psoriasis	IMO-3100	TLR7/8 inhibitor	NCT01622348
AGS	Baricitinib	JAK1/2 inhibitor	NCT01724580 and NCT03047369

### Treatments of autoimmune diseases

Targeted treatments for dsRNA-related autoimmune diseases focus on reducing the production or increasing the elimination of self-nucleic acid triggers, as well as blocking downstream IFN signaling pathways activated by these triggers. Although specific medications have not been approved for clinical use, several therapeutic strategies are currently being tested in clinical trials.

#### The removal of putative self-nucleic acid stimulation

Given that most immunostimulatory nucleic acids are generated through a reverse transcription step in the life cycle of endogenous retroelements, using reverse transcriptase inhibitors, previously utilized in HIV treatment, to limit the production of endogenous nucleic acids that drive aberrant IFN production has shown promise for patients with AGS (Rice et al., 2018). A clinical study involving individuals with various AGS genotypes demonstrated a significant impact of abacavir, lamivudine, and zidovudine on IFN signaling in AGS patients (NCT04731103) (Table 2).

#### Blocking RNA-sensing signaling

Preclinical evidence supports the potential therapeutic utility of RNA sensor antagonists in autoimmune and inflammatory diseases by targeting unregulated nucleic acid sensing and cytokine overproduction. In mouse lupus models, TLR7/8 inhibition with M5049 (Enpatoran), a small-molecule inhibitor, demonstrated efficacy (Vlach et al., 2021). A phase II clinical trial assessing M5049 (Enpatoran) in SLE and cutaneous lupus erythematosus (CLE) is actively recruiting participants (NCT05162586). Similarly, BMS-986256 (Afimetoran), a mixed TLR7 and TLR8 antagonist, is currently under evaluation for efficacy and safety in SLE patients (NCT04895696) after a clinical study on pharmacokinetics in healthy male participants (NCT04269356). IMO-3100, a combined TLR7 and TLR8 antagonist, showed promise in reducing plaque psoriasis thickness in a recent study (NCT01622348) (Table 2). However, specific RLR antagonists have yet to be developed.

Alternative approaches to blocking downstream signaling include antibodies against IFN or the type I IFN receptor, as well as inhibitors targeting downstream signaling components. Janus kinase (JAK) inhibition has shown promise in treating various type I interferonopathies (Sanchez et al., 2018), although it necessitates careful consideration of associated risks, including infection, anemia, lymphopenia, and thrombosis, to be used in the context of autoimmune diseases. The integration of human genetics and molecular understanding of type I IFN-related autoimmune diseases has paved the way for precision medicine strategies that match the aberrant innate immune signaling source with targeted therapies.

### Therapeutic applications in cancer

Traditional anticancer therapies, including radiotherapy and chemotherapy, primarily target disrupting critical cellular processes involved in the cell cycle, such as DNA and RNA synthesis, mitotic spindle formation, and specific oncogenic signaling pathways in cancer cells (Bracci et al., 2014). However, emerging evidence indicates that these conventional treatments also engage the host's innate immune system, particularly through innate immune sensing mechanisms (Xu et al., 2017). Here, we focus on the utilization of endogenous RNAs to activate RNA-sensing pathways as a novel approach in cancer therapies, encompassing radiotherapy, chemotherapy, and immunotherapy.

#### Radiotherapy

Radiotherapy stands as a key treatment modality for cancer, administered to over half of patients (Baskar et al., 2012). It exerts direct cytotoxic effects to induce tumor cell death, while also stimulating the release of DAMPs, including RNA. Takemura et al. (2014) showed that high-dose irradiation leads to the leakage of cellular RNA, triggering widespread cell death via the TLR3–TRIF–RIPK1 signaling axis. Additionally, irradiation causes short nuclear RNAs, such as U1 and U2, to translocate to the cytoplasm, facilitating the formation of RIG-I:RNA complexes and initiating downstream signaling cascades (Ranoa et al., 2016). Moreover, irradiation facilitates the release of mtRNA into the cytoplasm, thereby activating the RIG-I–MAVS pathway and subsequent IFN-I production, underscoring a potential role for mtRNA in radiotherapy (Tigano et al., 2021).

#### Chemotherapy

Both innate and adaptive immune systems play significant roles in enhancing the anticancer efficacy of certain chemotherapeutic agents. By inducing immunogenic cell death in tumors, specific chemotherapeutic drugs not only activate innate immune sensing pathways but also provoke tumor-specific adaptive T-cell responses. Moreover, emerging evidence highlights the vulnerability of certain cancer cells to immunological responses mediated by dsRNA sensors, leading to the exploration of RNA sensor agonists as potential anticancer therapies toward enhancing host immunosurveillance against tumors (van den Boorn and Hartmann, 2013). Several pharmacological RNA sensor agonists, such as imiquimod (a TLR7/8 agonist) and anthracyclines (a TLR3 agonist), have entered clinical development as reviewed elsewhere (Sistigu et al., 2014; Vanpouille-Box et al., 2019).

Furthermore, accumulating studies have revealed that traditional chemotherapy can induce epigenetic dysregulation in cancer, resulting in the accumulation of endogenous dsRNA and subsequent activation of dsRNA sensors and the IFN response, thereby improving treatment efficacy. CDK4/6 inhibitors (such as abemaciclib, palbociclib, and ribociclib) have been discovered not only to induce tumor cell cycle arrest but also to boost anti-tumor immunity (Patnaik et al., 2016). These inhibitors inhibit the E2F target DNMT1, leading to the transcriptional activation of ERV3-1 in tumor cells and the increased intracellular levels of dsRNA. Consequently, the RNA-sensing pathway in tumor cells is activated, resulting in the improved tumor antigen presentation (Goel et al., 2017).

Similarly, 5-azacitidine, a DNMTi approved for myelodysplastic syndromes and acute myeloid leukemia, induces bidirectional transcription of ERV-like genes, generating dsRNA and activating RNA-sensing pathways through a process known as ‘viral mimicry’ (Chiappinelli et al., 2015; Roulois et al., 2015). In various hematological malignancies, treatment with 5-azacitidine has led to the upregulation of three primary classes of REs (LINE, LTR, and SINE) and activation of the IFN pathway in responders, providing *in vivo* support for the role of viral mimicry as a crucial anti-tumor mechanism mediated by DNA demethylating drugs (Ohtani et al., 2020). Further clinical research is needed, particularly in solid malignant tumors. Regrettably, two completed clinical studies using 5-azacitidine did not meet the primary tumor response rate endpoint of response evaluation criteria in solid tumors (NCT01105377 and NCT01349959) (Azad et al., 2017). Currently, a phase 2 trial (NCT01928576) to investigate the priming effect of hypomethylating drugs on anti-PD1 antibodies is still underway.

#### Immunotherapy

Numerous RNA sensor agonists are in development as potential anti-tumor agents, many of which are being explored in conjunction with cancer vaccines or immune checkpoint blockade (ICB) therapy. For instance, inhibiting METTLE3 could induce dsRNA formation and trigger an IFN response, subsequently enhancing MHC-I antigen presentation and bolstering anti-tumor immunity (Guirguis et al., 2023). Particularly noteworthy is the combination of METTLE3 inhibition with anti-PD1 therapy, which has shown the enhanced efficacy in clinically relevant models of hematological and solid cancers (Yankova et al., 2021; Guirguis et al., 2023). Moreover, RNA sensor activation has demonstrated a potential in CAR-T cell therapy. For example, CAR-T cells engineered to express RN7SL1, an endogenous RNA that activates RLR signaling, have exhibited the capacity to promote the expansion of endogenous effector-memory and tumor-specific T cells, resulting in the rejection of solid tumors, even in scenarios where CAR antigen loss is present (Johnson et al., 2021).

Recent evidence also indicates a correlation between the expression of ERVs in tumor samples and markers of histone alterations and immune checkpoint pathways across various cancer types (Panda et al., 2018), suggesting that the effectiveness of ICB therapy, which depends on the expression of immune checkpoint genes, could be modulated by ERV transcription. Notably, the expression of ERV3-2 in patients with renal cell carcinoma has been linked to a positive response to PD-1 blockade (Panda et al., 2018). These findings support that endogenous RNA sensor ligands might serve as predictive markers for individual responses to ICB therapy (Smith et al., 2018).

In general, the activated RNA-sensing pathways during cancer treatment serve as adjuvants against cancer by transforming ‘cold’ tumors into ‘hot’ tumors and thereby enhancing anti-tumor efficacy.

## Conclusion and prospects

Recent research has significantly enhanced our understanding of RNA-sensing pathways activated by endogenous RNAs, covering a range of topics from mechanistic insights and pathogenesis to therapeutic potentials. However, several pivotal questions remain unresolved. A fundamental issue is how cells distinguish self-RNA from non-self-RNA in sterile environments. Given the diverse sources of cellular RNAs and the multiplicity of RNA-sensing pathways, it is evident that equally complex and varied regulatory mechanisms are in place at both levels of endogenous RNAs and RNA sensors to prevent their constitutive and aberrant activation. Although various regulators of dsRNA-sensing pathways have been identified, the precise orchestration among these regulatory strategies and their potential contributions to human diseases remain obscure. Additionally, different mutations within the same gene, and even identical mutations, can lead to varied disease types or severities. For instance, *Adar1*^E861A/E861A^ mice are lethal, while *Adar1*^W197A/W197A^ mice exhibit brain malformation—a key clinical feature in patients with AGS (Liddicoat et al., 2015; Nakahama et al., 2021); similarly, the R779H mutation in MDA5 can cause AGS or SLE in different individuals (Rice et al., 2014; Van Eyck et al., 2015). Further research is required to elucidate these genotype–phenotype relationships, essential for preclinical studies aiming at mitigating dsRNA-induced immunopathology. Moreover, the implications of chronic sterile activation of these RNA-sensing pathways on cellular senescence and aging remain underexplored, despite increasing evidence indicating that prolonged activation of the cGAS–STING pathway by mtDNA, which also induces IFN and inflammatory cytokine production, contributes to a senescence-associated secretory phenotype (De Cecco et al., 2019; Gulen et al., 2023; Victorelli et al., 2023).

In the realm of therapeutic development, RNA-sensing pathways have emerged as highly promising targets. Their distinct advantage lies in their ability to robustly enhance broader immune responses. This is primarily due to the abundance of RNA sensors, which are expressed across various cell types and can elicit a spectrum of effects including the production of IFNs and inflammatory cytokines, induction of the ISR, and even cell death. Particularly in oncology, augmenting RNA sensor activity can improve immune recognition of tumors, especially those adept at evading conventional detection by suppressing alternative immune pathways. This strategy holds potential for synergistic integration with existing immunotherapies, like checkpoint inhibitors, to boost their effectiveness. Nevertheless, the abundance of RNA sensors can also present a double-edged sword. The redundancy and overlap in multiple receptors and their signaling pathways, such as those seen in the roles of PKR, ZBP1, and MDA5 in the lethality associated with ADAR1 deficiency, could render single-target interventions ineffective, particularly in autoimmune diseases. This underscores the need for combination therapies, which may complicate treatment regimens and increase the risk of adverse effects. Furthermore, the long-term consequences of modulating RNA-sensing pathways remain poorly understood. Prolonged inhibition or chronic inflammation could potentially alter gene expression and immune cell function, raising the risk of infection and unforeseen adverse effects. Therefore, sequence optimization and chemical modifications have been pursued to temper excessive activation of RNA-sensing pathways, aiming to maintain appropriate immunogenicity in gene therapy and vaccination.

Overall, RNA-sensing pathways are critical not only for defending against pathogens but also for maintaining immune homeostasis; their aberrant activation can precipitate autoimmune diseases or be exploited to combat pathogens and cancer. The dual nature of RNA-sensing activation in disease management underscores the importance of judicious evaluation in diverse clinical scenarios and disease progression stages. As the exploration of RNA-sensing pathways in autoimmunity expands, meticulous research and strategic application will be essential to leverage their therapeutic potential while minimizing unintended effects.

## Notes

**Author contributions:** J.L. and J.Z. drafted the manuscript; H.Y. and F.H. revised the manuscript.
